# Static and Dynamic Measurement of Ocular Surface Temperature in Dry Eyes

**DOI:** 10.1155/2016/7285132

**Published:** 2016-06-28

**Authors:** Li Li Tan, Srinivasan Sanjay, Philip B. Morgan

**Affiliations:** ^1^School of Chemical and Life Sciences, Singapore Polytechnic, Singapore 139651; ^2^Ophthalmology and Visual Sciences, Khoo Teck Puat Hospital, Singapore 768828; ^3^Yong Loo Lin School of Medicine, National University of Singapore, Singapore 119228; ^4^Eurolens Research, The University of Manchester, Manchester M13 9PL, UK

## Abstract

*Purpose*. To study ocular surface temperature (OST) in dry eyes by static and dynamic measures.* Methods*. OST was recorded on 62 dry eyes and 63 age- and sex-matched controls. Static measures were study of absolute OST at *t* = 0, 5, and 10 s after eye opening. Dynamic measures were study of mean change and net change in OST over 10 s of sustained eye opening. Ten OST indices studied were temperatures of the geometric center of the cornea (GCC), extreme temporal (T1) and nasal conjunctiva (T4), midtemporal (CT) and nasal conjunctiva (CN), temporal (LT) and nasal (LN) limbus, and mean (MOST), maximum (Max *T*), and minimum (Min *T*) temperatures of the region of interest.* Results*. For static measures, dry eyes recorded significantly lower GCC, MOST, Min *T*, Max *T*, T4, CT, LT, LN, and CN. For dynamic measures, dry eyes had significantly steeper regression line of mean change (corresponding to greater net change) for Max *T* 5 s onward and T4 at 3 s onward.* Conclusions*. Both static and dynamic measures of the OST were valuable and can be used as clinical tool to assess dry eye.

## 1. Introduction

Dry eye disease (DED) is multifactorial and can be caused by poor quality tear film and inflammation of the eyelid/ocular surface [1] resulting from a lower tear production rate and/or a tear instability. In DED, the ocular surface becomes dry and lacks lubrication, eventually leading to ocular surface damage [2]. Infrared (IR) ocular thermography determines ocular surface temperature (OST) of the eye and preorbital skin by measuring the amount of IR radiation emitted from the surface with an infrared thermal imaging camera. Measurements are then processed into a color coded display image (thermogram) for interpretation and analysis [3]. Noninvasive ocular thermography was first introduced in 1968 and was used to evaluate both normal and pathological conditions [4–7].

Capturing OST changes using IR ocular thermography reflects the nature of the tear film and its stability [8–10] and has been used to study DED since 1993 [3]. Morgan and his associates [3] investigated the temperature difference between limbus and central cornea (termed as RTD, radial temperature difference) on a 66-year-old chronic dry eye patient and reported a significant difference in RTD between dry eye (1.40°C) and healthy eye (0.37°C). Further studies on dry eye have been performed using ocular thermometry/thermography since then [3, 8, 11–22] and the findings are summarised in Table 1. Most of the reported studies only considered static measures (i.e., study of OST on a single frame) or temporal/dynamic measures (i.e., study of OST changes over time). The OST indices studied were limited and did not document the whole exposed ocular surface. Software used and method employed to precisely define anatomical locations across the ocular surface have also varied (Table 1). Our study was designed to investigate the ocular surface temperature in dry eye using both static and dynamic measures. A novel “diamond” method in marking the ocular surface and OST acquisition was employed [23] and ten OST indices were included to document the whole inferior zone of the exposed ocular surface.

## 2. Methods

### 2.1. Subjects

The research protocol was approved by the Singapore National Health Group (NHG) Domain-Specific Review Board (DSRB) and the Singapore Polytechnic ethics review committee and the work adhered to the tenets of the Declaration of Helsinki. Informed consent was obtained from each subject at study enrolment. Sixty-two dry eye (48 ± 10 years; 14 male and 48 female) and 63 age- and sex-matched control subjects (46 ± 7 years; 16 male and 47 female) completed the study. The inclusion criteria for the dry eye subjects were as described previously [23]: use of tear replacement therapy and having either a fluorescein tear break-up time of 10 seconds or less [24] or a Schirmer I test result of less than 10 mm in 5 min [11] along with presence of corneal or conjunctiva staining. All dry eye patients were screened and diagnosed by an ophthalmologist at Khoo Teck Puat Hospital eye clinic. Classification of mild or moderate and severe patients was based on a composite disease severity index, derived from the Dry Eye Workshop severity scale [2]. Control subjects were those not using tear replacement therapy or any topical medication and without signs or symptoms of dry eye. All subjects were required to be noncontact lens wearers for at least two years prior to enrolment. Subjects were excluded from control group if they had Schirmer I test result of less than 10 mm in 5 min or fluorescein tear break-up time of 10 seconds or less. Subjects with any anterior ocular anomalies (e.g., current ocular infection, allergy, or ptosis), those who had undergone surgery or are taking any medication that could affect the tear film, or those who were currently pregnant or breastfeeding were excluded [23].

### 2.2. Procedures

The procedures were the same as described previously [23]. Subjects refrained from using their eye-drops or eye make-up on the day of measurement. Ocular thermography was performed in real time using an infrared thermotracer (NEC TH9420) with resolution of 640 (H) × 480 (V) pixels, operational sensitivity of 0.06°C, and frequency of 30 frames per second, detecting infrared radiation between 8 and 14 *μ*m. The emissivity of 0.98 was assumed [7]. A standard examination protocol as reported in the literatures [8, 10, 11, 17] was adopted. All the measurements were performed from 9 am to 2 pm in the same room with controlled room temperature (24.06 ± 0.41°C) and humidity (49.76 ± 2.61%), with no air drifts and the same brightness (380 lux). Subjects were adapted to the room for 20 minutes prior to ocular thermography as previous work has shown that this period was necessary to achieve ocular temperature stabilisation [25]. As corneal temperature is strongly associated with body temperature and seemed to plateau at 36.5°C to 37.0°C [26], body temperature for all subjects was measured and subjects with body temperature ≥37°C were excluded. OST was recorded under the conditions described by Morgan and associates: the subjects blinked normally, closed for 3 s, and the first image was recorded just after the eyes had opened [11, 27]. 0 s was recorded as the time upon eye opening. 300 frames of real time thermal images reflecting OST changes at the ocular surface were captured over 10 s sustained eye opening. The measurement was done three times on the right eye followed by the left eye. At any time if subject blinked or changed fixation before 10 s, the measurement was discounted and repeated.

**Figure 1:**
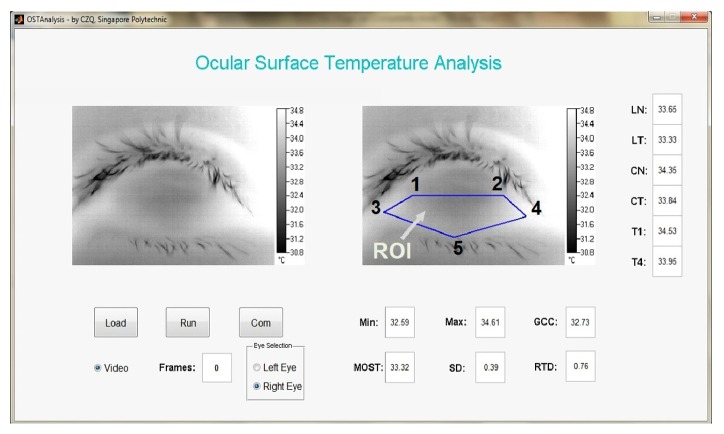
Ocular surface marking and OST acquisition using the novel “diamond” method.

A novel “diamond” method was used to mark the ocular surface using a custom-designed OST Analysis V2 software (developed using MatLab Simulink 7.11.0, R2010b). The region of interest (ROI) formed by five anatomical points across the ocular surface (labelled as 1–5) was shaped like a diamond (Figure 1). This method has the advantages of (1) overcoming reported problems of truncated image by upper lids [28], (2) minimizing possible inconsistency in OST acquisition due to variation in palpebral aperture size, and (3) enabling study of the inferior zone of the ocular surface that was reported to be a predictive area in detection of dry eye subtypes [29]. Each point marked represents an area of 3 × 3 pixels so that temperature was an average of nine pixels:

1. Temporal limbus (LT).
2. Nasal limbus (LN).
3. Extreme temporal conjunctiva (T1).
4. Extreme nasal conjunctiva (T4).
5. Most inferior point of the ocular surface.

Once the marking of ROI was completed, OST acquisition and processing was performed automatically by double clicking the last point marked (point 5) to activate the OST Analysis V2 program and process all the 300 frames. All frame marking and data processing were undertaken by a single examiner (LL). Ten OST indices of the ocular surface were generated as shown in Table 2. In this study, GCC denotes the temperature of the geometric center of the cornea, obtained midway between LT and LN. The OST indices were selected to document the whole inferior zone of the exposed ocular surface within ROI which had included most of the reported OST indices as shown in Table 1 with few newly added indices. All the ten OST indices extracted by the “diamond” method were shown to be highly repeatable in assessing healthy and dry eyes [23] in terms of interimage, interexaminer, and intraexaminer variability.

### 2.3. Statistical Analysis

Static measures were the study of absolute OST at *t* = 0 s, 5 s, and 10 s after eye opening. Dynamic measures were the study of mean change (relative to baseline) as well as net change in OST over 10 s of sustained eye opening. To prevent difficulties arising when nonindependent data were collected from both eyes, only data obtained from right eye were used in the analysis [30]. Statistical analyses were performed using JMP version 12.1.0 (http://www.jmp.com/; SAS Institute Inc., USA). For static measures, one-way ANOVA was performed for the ten indices studied to explore differences between dry eye and control subjects at *t* = 0 s, 5 s, and 10 s. For dynamic measures, a general linear model was constructed (two-way repeated measures ANOVA model) to test the significance of each group, time, and their interaction (group by time) effects, where the interaction between groups over time was the key outcome. Post hoc analysis for significance of the group effect at each time point was conducted using unpaired *t*-test (two-tailed), on OST indices with significant outcomes. All tests were two-tailed and *p* < 0.05 was considered significant. All data were presented as mean ± SD, unless otherwise stated.

## 3. Results

All dry eye subjects were mild to moderate with no inflamed meibomian glands. We acknowledged that many disease severity criteria are confounded by complex disease subtypes and a lack of standardisation, and the selection of single criteria for assessment of disease severity is therefore fraught with difficulties [31–33]. Both dry eye and control subjects showed positive compliance during ocular thermography measurements and therefore it was possible to record reliable data in all cases. There were no reports of reflex tearing and any discomfort from the subjects during the course of the study. Although eight seconds of sustained eye opening has been reported to be an easily achievable target for subjects without causing reflex tearing [10], other studies had reported to be equally achievable for subjects to hold for 10 s [14, 17].

**Figure 2:**
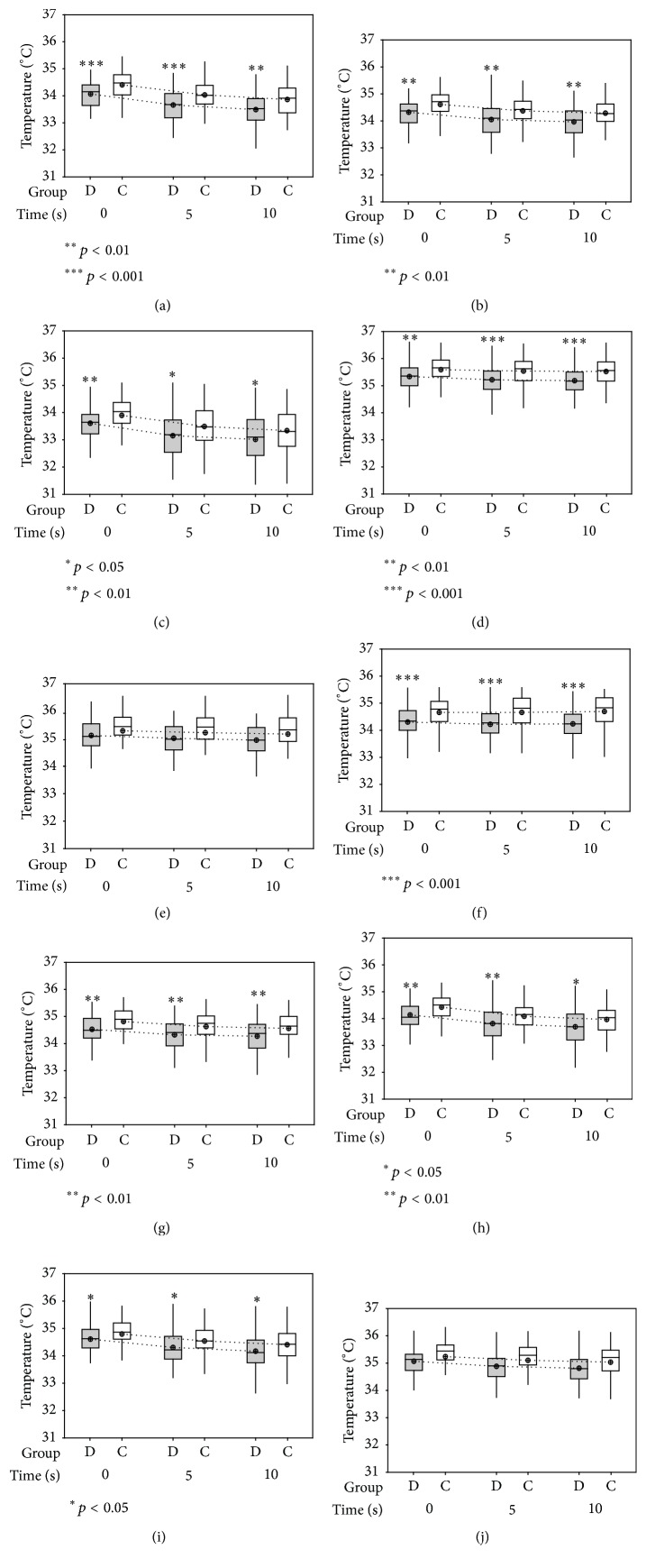
*(Static measures)* box plots showing comparison of absolute OST at 0 s, 5 s, and 10 s: (a) GCC; (b) MOST; (c) Min *T*; (d) Max *T*; (e) T1; (f) T4; (g) CT; (h) LT; (i) LN; and (j) CN in (grey box) dry eye subjects and (white box) controls. The results were expressed as median and mean ± SD. Mean-connecting-lines are represented by dotted lines to show the change in mean over 0 s, 5 s, and 10 s. *p values* are shown using one-way ANOVA at 95% CI.

For static measures, dry eye recorded a significantly lower temperature (for GCC, MOST, Min *T*, Max *T*, T4, CT, LT, and LN) as compared to controls at 0 s, 5 s, and 10 s (one-way ANOVA, *p* < 0.05). The differences were highly significant (*p* < 0.01) for GCC, MOST, Max *T*, T4, CT, and LT and were significant (*p* < 0.05) for Min *T* and LN. There were marginal significant differences found for CN at 5 s and 10 s and no significant differences were found between the two groups for T1 (Figure 2).

**Figure 3:**
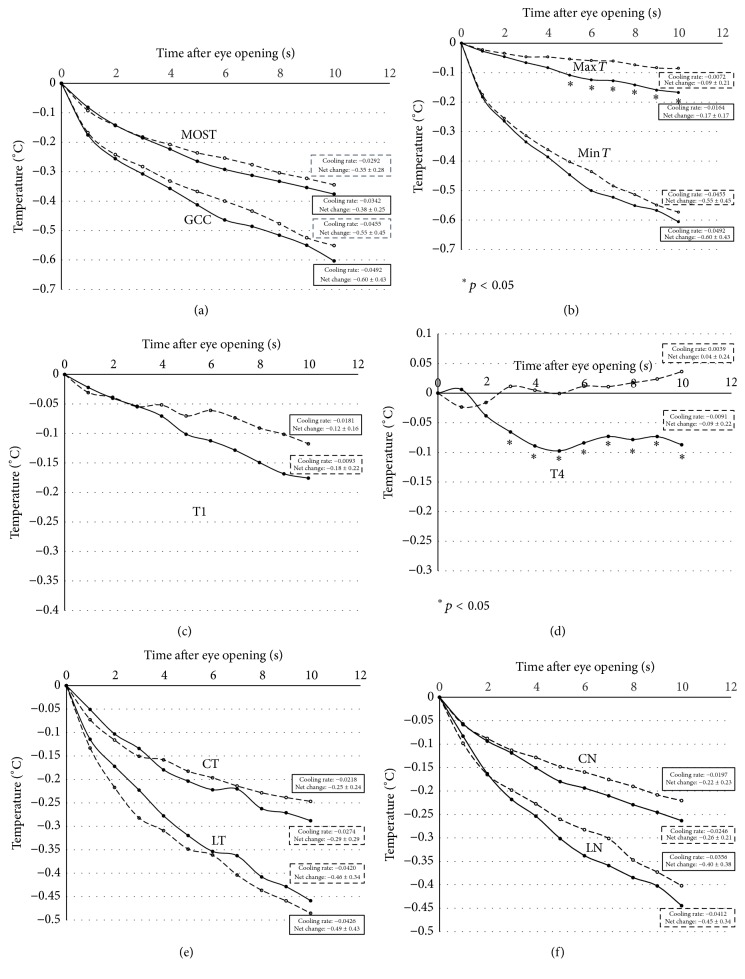
*(Dynamic measures)* graphs showing the mean change OST (relative to baseline) during the 10 s sustained eye opening: (a) GCC and MOST; (b) Min *T* and Max *T*; (c) T1; (d) T4; (e) CT and LT; (f) CN and LN in (solid circles) dry eye subjects and (open circles) controls. Values in boxes represent the cooling rate and net change in OST over the 10 s period in dry eye and control groups, respectively. A comparison of mean at each 1 s interval was performed using unpaired *t*-test, ^*∗*^ *p* < 0.05. A typical standard deviation for GCC was ±0.29 (= average std deviation) for dry eye and ±0.30 for controls and for MOST it was ±0.15 for dry eye and ±0.18 for controls. A typical standard deviation for Min *T* was ±0.33 for dry eye and ±0.32 for controls and for Max *T* it was ±0.11 for dry eye and ±0.13 for controls. A typical standard deviation for T1 was ±0.14 for dry eye and ±0.11 for controls and for T4 it was ±0.21 for dry eye and ±0.16 for controls. A typical standard deviation for CT was ±0.19 for dry eye and ±0.17 for controls and for LT it was ±0.23 for dry eye and ±0.29 for controls. A typical standard deviation for CN was ±0.14 for dry eye and ±0.16 for controls and for LN it was ±0.22 for dry eye and ±0.23 for controls.

For dynamic measures, dry eye had significantly steeper regression line of mean change (corresponding to greater net change) only for two out of the ten OST indices: Max *T* and T4 (Figure 3). Two-way analysis of variance showed that there were significant group by time interaction effects for Max *T* and T4 temperatures (Max *T*: *F* = 4.6814, *p* = 0.0324; T4: *F* = 5.9506, *p* = 0.0161). For Max *T*, the drop in mean change in dry eye was statistically significant from 5 s onward (unpaired *t*-test, 5 s, *p* = 0.037; 6 s, *p* = 0.012; 7 s, *p* = 0.022; 8 s, *p* = 0.023; 9 s, *p* = 0.016; 10 s, *p* = 0.019). Net change for Max *T* in dry eye over 10 s was −0.17 ± 0.17°C, which was two times greater as compared to controls (−0.09 ± 0.21°C). Cooling rate as indicated by gradient of the graph for Max *T* was also twice as much as in dry eye (−0.0164°C/s) as compared to controls (−0.0072°C/s) (Figure 3). For T4, the drop in mean change has only happened in dry eye group and it was statistically significant from 3 s onward (unpaired *t*-test, 3 s, *p* = 0.023; 4 s, *p* = 0.005; 5 s, *p* = 0.014; 6 s, *p* = 0.009; 7 s, *p* = 0.047; 8 s, *p* = 0.036; 9 s, *p* = 0.028; 10 s, *p* = 0.005). T4 for control group was pretty stable during the 10 s of sustained eye opening. Net change for T4 in dry eye over 10 s was −0.09 ± 0.22°C, which was more than two times greater as compared to controls (−0.04 ± 0.24°C). Cooling rate for T4 was also more than two times greater in dry eye (−0.0091°C/s) as compared to controls (−0.0039°C/s). No significant differences were found in mean change at any point of time (unpaired *t*-test at each 1 s interval, *p* > 0.05) between the two groups for other OST indices during dynamic measures (Figure 3).

## 4. Discussion

The current study demonstrated the ability of IR ocular thermography in assessing dry eye. Ten OST indices were evaluated in two aspects: static and dynamic measures. Each OST index studied represented an area of 3 × 3 pixels, except for MOST. Rather than report on individual pixel values which might be subject to local variation, we selected 3 × 3 pixels and took the average temperature. We believe this is good compromise between single pixels and a larger ROI which would provide less opportunity to analyse specific geographic areas of interest.

OST in dry eye was different from controls at different ocular surface areas during static measures upon eye opening (*t* = 0 s) as well as when *t* = 5 s and 10 s. As compared to controls, the ocular surface of dry eye subjects was significantly cooler as recorded at the geometric center of the cornea (GCC) as well as various areas at conjunctiva (T4, CT, and CN) and limbus (LT and LN) and causing an overall lower mean ocular surface temperature (MOST). As the ocular surface measured by the thermotracer consists of cornea-conjunctiva-limbal complex, it was not surprising to record a significantly lower minimum temperature of the ocular surface (Min *T*) and maximum temperature of the ocular surface (Max *T*) in dry eye. During dynamic measures, OST was found to drop over the 10 s of sustained eye opening in both the dry eye and control subjects. Only two OST indices (out of ten) had significant steeper regression line of mean change with greater net change in dry eye. As for the temperature of the extreme nasal conjunctiva (T4), the change was only observed in dry eye subjects and was statistically significant from 3 s onward.

### 4.1. Static Measures

The primary source of ocular radiation measured by ocular thermography is the tear film [11] so changes in tear film thickness and its composition alter the temperature measured [8]. Lower OST in the dry eye group found in the current study could be due to a thinner tear film as a result of a thinner tear film lipid layer (TFLL) in dry eye [34, 35]. TFLL has been reported to be important in tear film stability and evaporation [36] and is abnormal in dry eye [8, 37]. According to the Dry Eye Workshop report [2], tear film instability is one of the two core mechanisms of dry eye and can lead to thickness variation and an overall thinner tear film. The ocular surface was cooled by a thinner tear film leading to lower temperature recorded on various ocular surface areas (geometric center of the cornea, conjunctiva and limbus) during static measures at *t* = 0 s. OST was likely to be affected by variations in evaporation in the seconds after eye opening, in addition to the effects of convection [8]. Not unexpectedly, lower temperatures were noted in dry eye subjects after 5 s and 10 s of eye opening. Thermography has been reported as an indirect method to evaluate tear evaporation rates and tear film impairment due to its ability to record subtle changes over the corneal temperature [14]. Indeed, tear evaporation in dry eye can cause a 10-fold reduction in tear thickness after a blink [38] and was found to be correlated with lower corneal temperature and subjective discomfort symptoms [22]. This was apparent in the current report revealing a declining temperature on the various ocular surface areas from 0 s to 10 s during static measures. The temperature gradient is varying at different OST indices and suggestive of different evaporation rate at different areas of the ocular surface. Lower OST can also be accounted for by the presence of “cold receptors,” a class of ion channels identified in nerve endings and in corneal and conjunctival epithelial cells that can mediate the pain transduction from the ocular surface [39]. Although temperature variation has been reported to be higher in dry eyes when RTD (radial temperature difference) was studied by Morgan et al. [3, 11], RTD was not included in the current study as previous report [23] has shown poor RTD repeatability when measured using the current thermotracer.

A lower MOST value in dry eye was in agreement with the report by Singh and Bhinder [16, 40] using remote sensor thermometry but conflicts with the report by Morgan et al. [11] and Singh and Bhinder [41] using IR thermography and IR thermometry, respectively. A warmer overall ocular surface has been accounted for by the increased conjunctival hyperaemia in dry eye [11, 41]. The vascularised conjunctiva is an important heat source [3] to the ocular surface. Certainly, OST is increased during inflammatory disease [3, 5] and ocular surface inflammation is a core mechanism in dry eye [42]. A higher MOST could also be associated with higher blink rate in dry eye [43]. The conflicting results found in our study as compared to those previously reported may be due to various reasons. Firstly, most of our dry eye subjects did not present with conjunctival hyperaemia, the results were therefore different. Secondly, different experimental methodologies may lead to different findings as suggested by Kamao et al. [17]. The “diamond” method in marking the ocular surface and OST acquisition in our study allowed a more holistic study on MOST as it covers a wider area of the ocular surface as compared to obtaining the MOST by averaging the temperature of the cornea and conjunctiva across the horizontal meridian in Morgan et al.'s [11] study. Furthermore, the ROI studied was the lower half of the ocular surface whereby tear film will thin faster [29] as compared to other areas due to evaporation and leads to a lower MOST in dry eye as shown in the current study. OST measurements by Singh and Bhinder [41] were made in a closed chamber instead of an open atmosphere so comparison with that work is clearly problematic as local environmental factors influence OST [44]. Thirdly, the severity of dry eye varied by report. Dry eye subjects recruited by Morgan et al. [11] were mostly severe dry eye cases whereas the dry eye subjects in our study ranged from mild to moderate cases. In more severe cases, the level of local inflammation and greater conjunctiva hyperaemia may overwhelm evaporative effects, leading to a warmer ocular surface [11]. Last but not least, age of subjects recruited could also cause different results. In previous projects, there was no apparent attempt to age-match the dry eye and the control groups. This is important because OST was reported to decrease with age at a rate of −0.010°C/year [27] and the dry eye populations used by previous reports [11, 14, 17, 18] were much older compared to the controls; in other words, differences between the groups may be age-related rather than related to the disease itself.

### 4.2. Dynamic Measures

During dynamic measures, our results were in agreement with literatures that ocular surface cooled during sustained eye opening [12, 45] and rate of the cooling was greater in the dry eye group [12]. This was also in consistent with a mathematical model developed by Peng et al. [46], which postulates a mechanism by which local rupture of the TFLL increases local tear evaporation rate leading to tear film rupture and tear film break-up. Tear film thinning and break-up have been shown to correspond to ocular surface cooling over time [45]. Dry eye had a thinner TFLL [34, 35] and, upon eye opening, the tear film starts to thin due to evaporation leading to drop in temperature [36]. The tear film lipid could have depleted and no longer hold/protect the aqueous layer at *t* = 5 s (for Max *T*) and at *t* = 3 s (for T4) and hence a sudden increase in evaporation/tear film thinning and ocular surface cooling. The cooling rate for Max *T* was twice as much as in dry eye as compared to controls and may indirectly reflect the rate of evaporation in dry eyes as reported by Li et al. [45]. A twofold [47] and a threefold [48] increase in the tear evaporation rate in dry eyes has been reported previously. Although tear evaporation was not measured in the current study, tear film cooling in dry eye upon eye opening as a result of tear evaporation as well as greater effect of the positive latent heat of tear vaporisation has also been reported [8, 12, 49].

Changes in T4 were only observed in dry eye subjects and could be easily differentiated from their controls (Figure 3). Conjunctival temperature was reported to be higher than the central cornea [7, 50]. Although the reasons remain unclear, temperature of the nasal conjunctiva was reported to be higher than that of the temporal conjunctiva because of the influence of greater blood flow due to more large vessels including the dorsal nasal artery and the angular artery at the nasal conjunctiva [17]. There are more large vessels, including the dorsal nasal artery and the angular artery, on the nasal side of the eye, and the medial rectus muscle has two anterior ciliary arteries, whereas the lateral rectus muscle has only one artery [17]. The difference in vascularisation at nasal and temporal conjunctiva could have created different tear film cooling rate in these two areas upon eye opening and therefore the different results found in T4 and T1. The reason why there was no change in T4 for the controls warranted further investigations. Studying T4 at 3 s and onwards can be a potential diagnostic index for dry eye due to its “unique” behaviour as compared to non-dry eye subjects. Based on our findings, 10 s of sustained eye opening may not be required as it is hard for dry eye patients to keep their eyes open for 10 s without inducing reflex tearing and blinking.

## 5. Conclusions

Static and dynamic measurement of the OST provided two different aspects in studying the tear film. Both measurements were useful and can be used as clinical tool to assess dry eye.

## Figures and Tables

**Table 1:** Studies on dry eye using ocular thermography reported in the literatures.

Authors	Subjects (mean age)	Thermotracer used (specification)	Ocular surface marking and OST acquisition	OST indices studied	Static/dynamic (time upon eye opening)
Morgan et al. [3]	1 D (66 yo)	IR thermographer NEC6T62 (Sensitivity 0.1°C Freq: 1 frame/s Resolution: 10 × 10 pixels)	—	RTD	Static

Morgan et al. [11]	36 D (58 yo) 27 C (57 yo)	IR thermographer NEC6T62 (Sensitivity 0.1°C Freq: 1 frame/s Resolution: 10 × 10 pixels)	Five 10 × 10 pixels placed in 5 anatomical locations along horizontal meridian running across centre cornea	MOST RTD	Static

Morgan et al. [12]	11 D (50 yo) 7 C (53 yo)	IR thermographer NEC6T62 (Sensitivity 0.1°C Freq: 1 frame/s Resolution: 10 × 10 pixels)	4 mm^2^ area at the centre of cornea	GCC	Dynamic (7 s)

Fujishima et al. [13]	20 D (37.9 yo) 20 C (35.1 yo)	IR radiation thermometer THI-500 (Sensitivity: 0.1°C Freq: 1 frame/s)	Central cornea	GCC	Static and dynamic (10 s)

Mori et al. [14]	13 D (45.5 yo) 7 C (33.6 yo)	Thermal Vision Laird 3 (Nikon) (Sensitivity: 0.15°C Freq: 60 frames/s)	20 × 20 pixel box at central cornea	*K* value	Dynamic (more than 10 s)

Craig et al. [8]	8 D (60.3 yo) 13 C (24.8 yo)	IR thermographer NEC6T62 (Sensitivity 0.1°C Freq: 1 frame/s Resolution: 10 × 10 pixels)	Mean of central cornea pixels	GCC TVF	Static

Zelichowska et al. [15]	9 D 13 C	IR radiation thermographer	Central cornea	GCC	Static and dynamic (15 s)

Singh and Bhinder [16]	51 D (35.36 yo) 51 C (35.36 yo)	IR and remote heat sensor thermometry (Sensitivity 0.1°C)	Closed and open eye temperature for 5 s	MOST	Static

Kamao et al. [17]	30 D (52.9 yo) 30 C (42.7 yo)	Tomey IR thermographer (Sensitivity 0.1°C Freq: 6 frame/s Resolution: 320 × 240 pixels)	Central cornea (GCC) 4 mm in diameter, CT and CN (both 2 mm in diameter)	GCC CN CT	Static and dynamic (10 s)

Su et al. [18]	76 D (49 yo) 47 C (34 yo)	Microbolometer sensor (Sensitivity 0.1°C Freq: 30 frame/s Resolution: 320 × 240 pixels)	ROI (region of interest) determined by four curves connected between four manually set apexes (top and bottom of the eye, left and right corners of the eye)	TDV CV	Dynamic (6 s)

Kottaiyan et al. [19]	—	Thermovision A40 (Sensitivity 0.08°C Freq: 30 frame/s Resolution: 320 × 240 pixels)	Central corneal	—	Dynamic (5 s)

Azharuddin et al. [20]	42 D (35.2 yo) 36C (28.4 yo)	FLIR SC305 (Sensitivity < 0.05°C Freq: 9 frame/s Resolution: 320 × 240 pixels)	Cornea	—	Dynamic (15 s)

Zhang et al. [21]	20 D (55.8 yo) (5 ADDE) (15 MGD)	FLIR SC325 (Sensitivity < 0.05°C Freq: 30 frame/s Resolution: 320 × 240 pixels)	Central cornea 7~9 mm circular	—	Dynamic (5 s)

Versura et al. [22]	24 D (52 yo) 15 C (43 yo)	Tomey TG 1000	Central cornea 4 mm circular	CCT	Static

D: dry eye subjects; C: control subjects.

RTD: radial temperature difference; MOST: mean ocular surface temperature; GCC: geometrical centre cornea.

TVF: temperature variation factor; CN: nasal conjunctiva; CT: temporal conjunctiva.

TDV: temperature difference value (temporal variation of OST); CV: compactness value (spatial variation of OST); *K* value: steepening of corneal temperature.

ADDE: aqueous deficient dry eye; MGD: meibomian gland dysfunction.

CCT: central corneal temperature.

**Table 2:** The ten OST indices studied.

OST indices	Description	Remarks
GCC	Geometric center of the cornea (midway between LT and LN)	Most commonly studied in the literature

MOST	Mean OST of the ROI	—

Min⁡*T*	Minimum temperature of ROI	Study of the minimum and maximum temperature of the ocular surface
Max⁡*T*	Maximum temperature of ROI	

T1	Extreme temporal conjunctiva	Study of the different areas of the limbus and conjunctiva
T4	Extreme nasal conjunctiva	
CT	Midtemporal conjunctiva (midway between T1 and LT)	
LT	Temporal limbus	
LN	Nasal limbus	
CN	Midnasal conjunctiva (midway between T4 and LN)	
